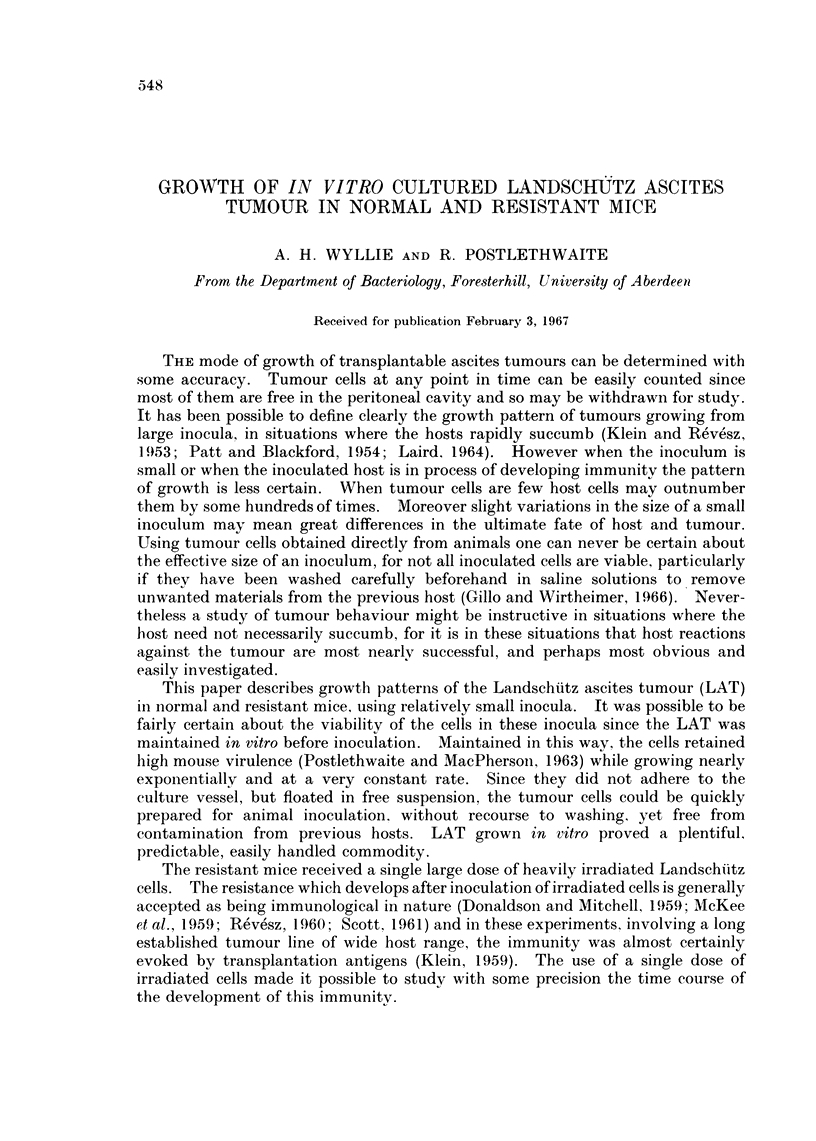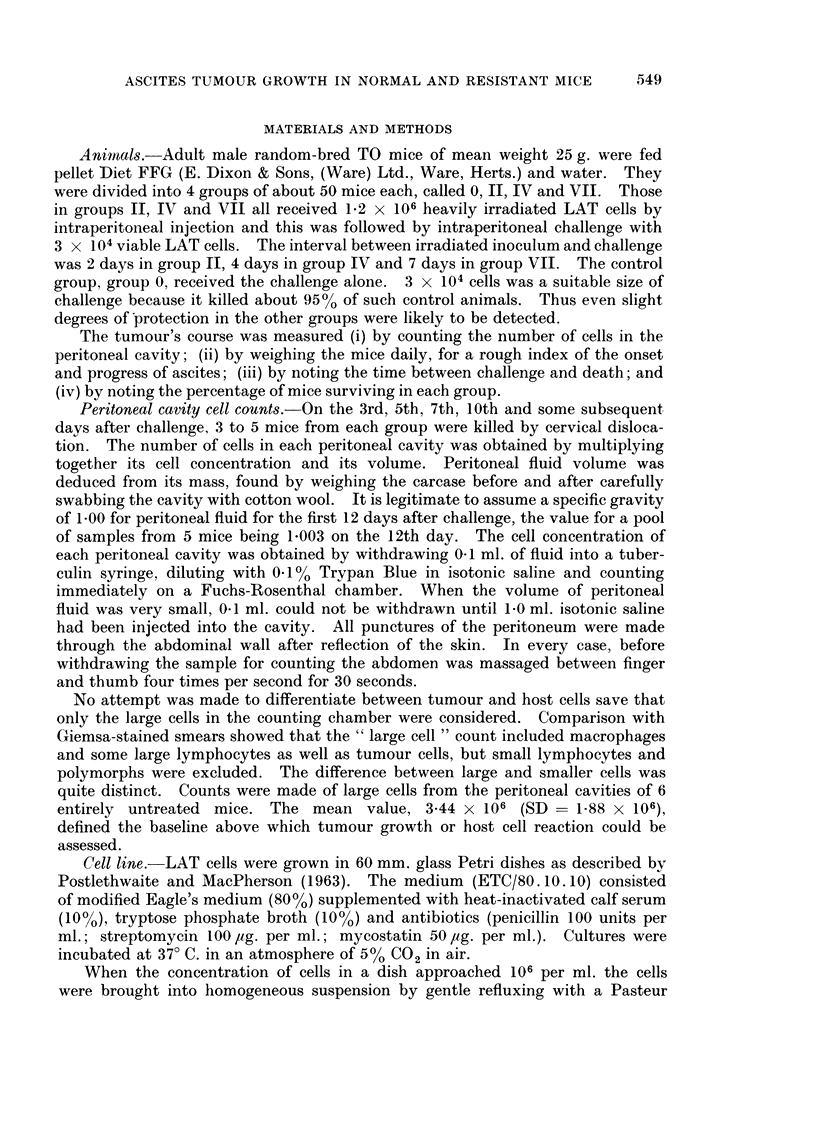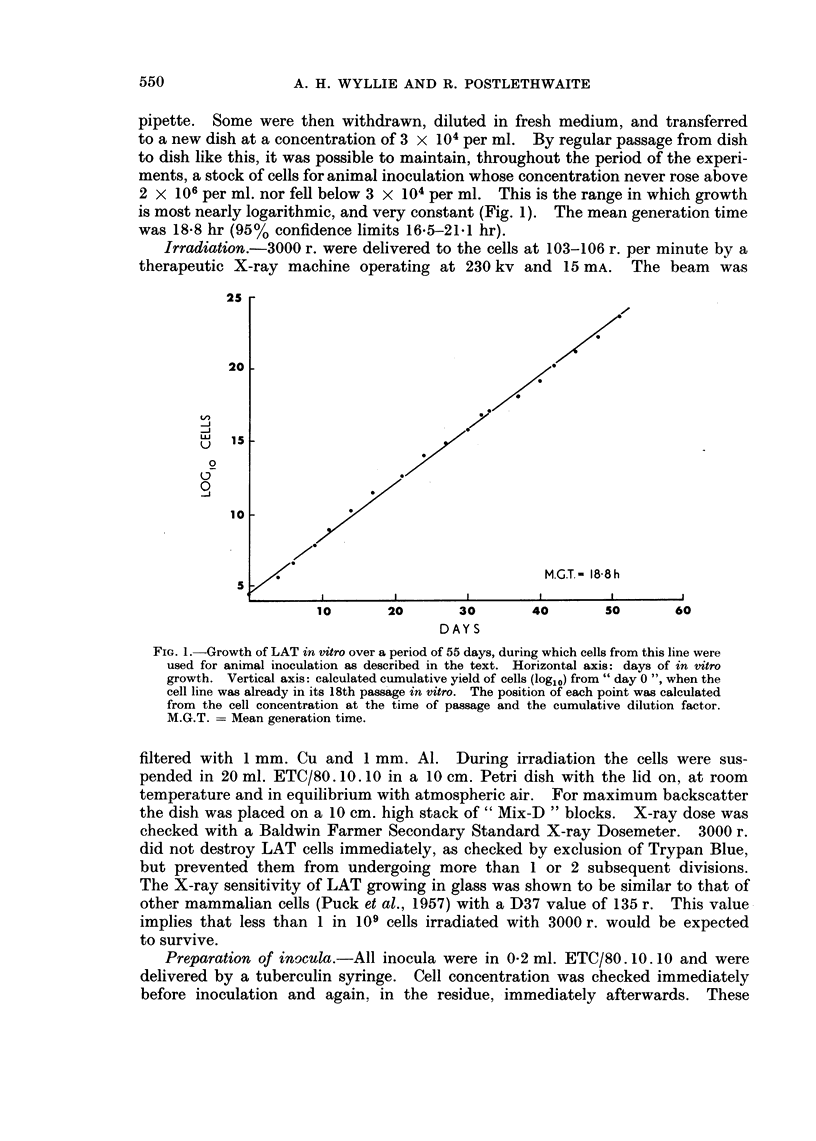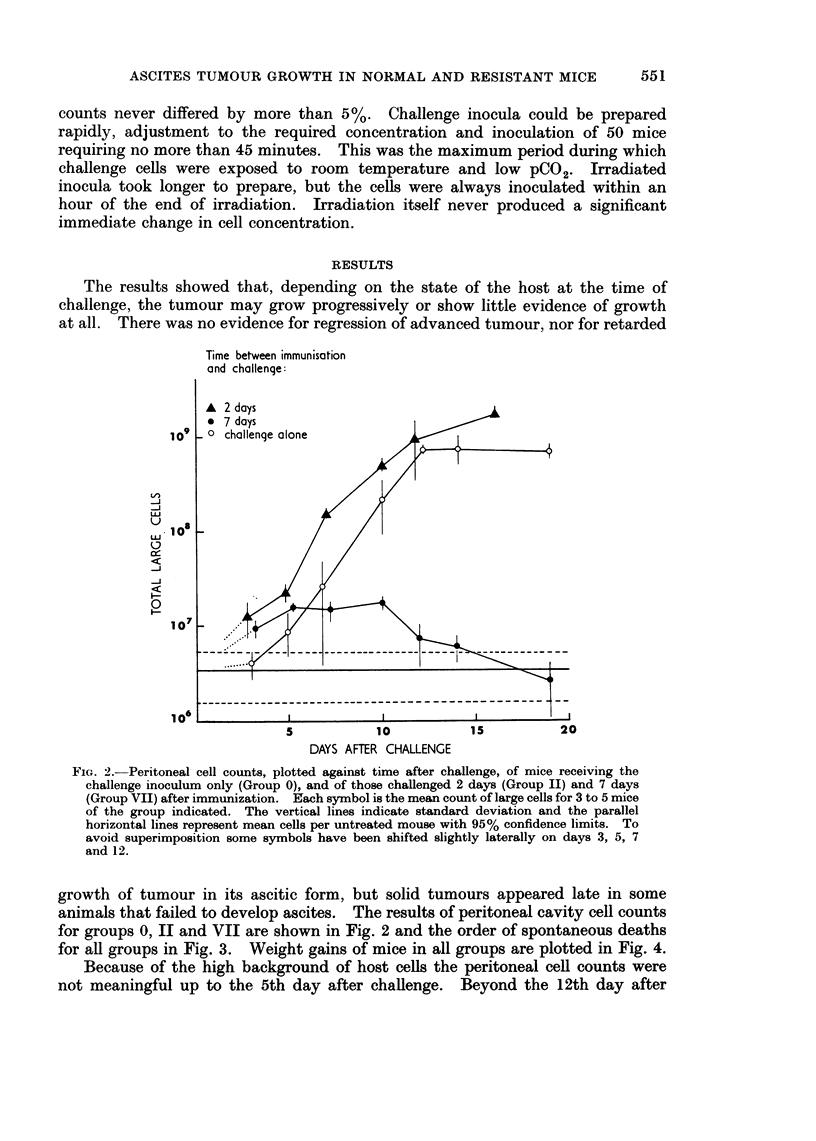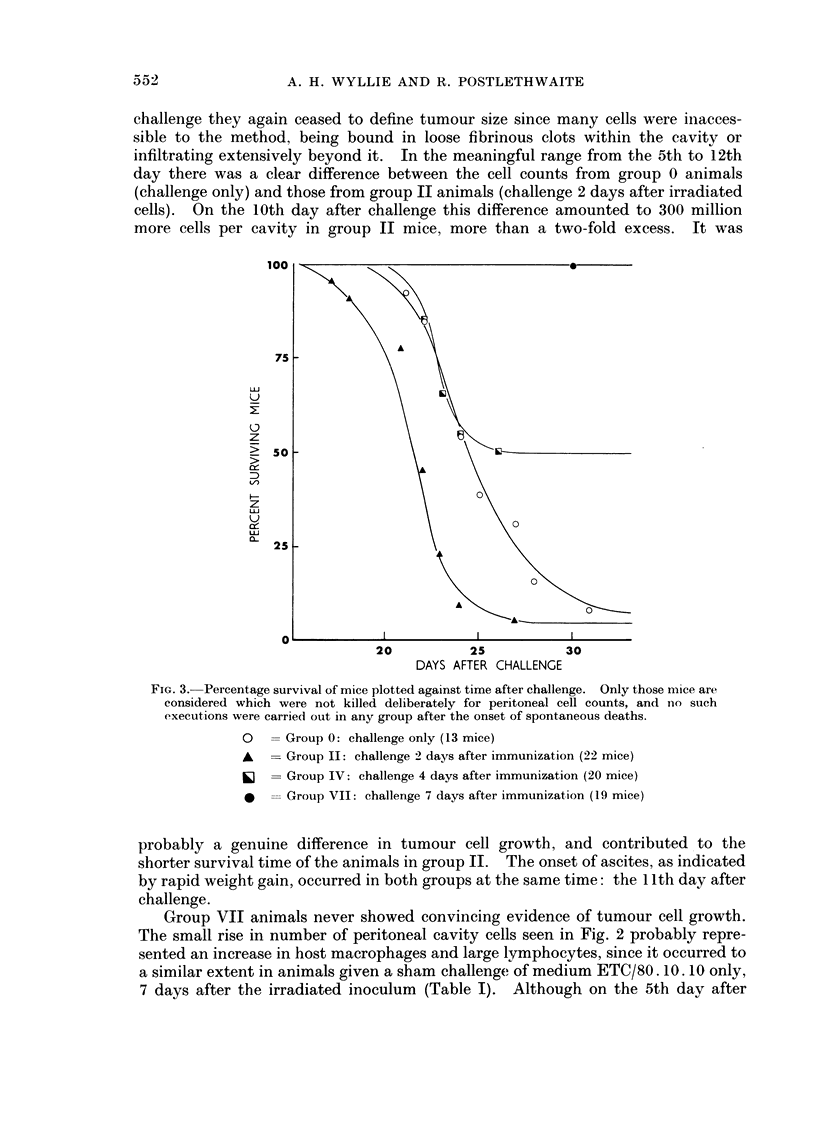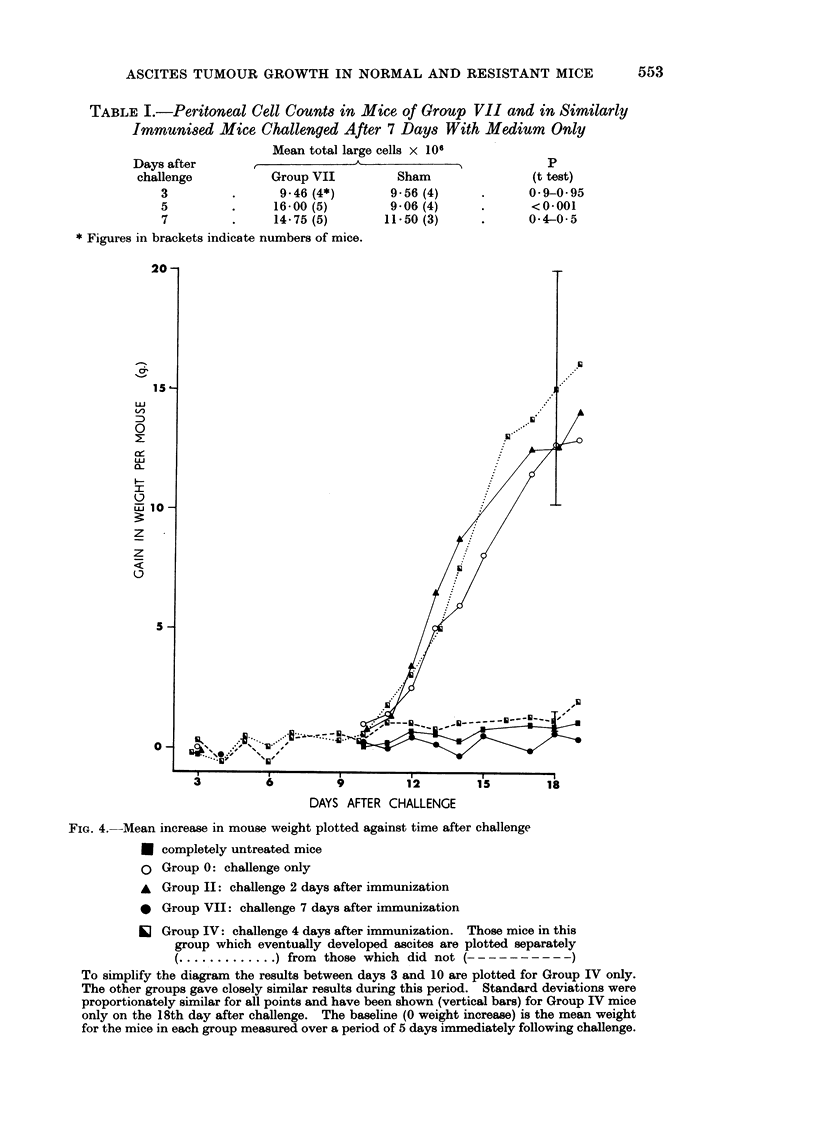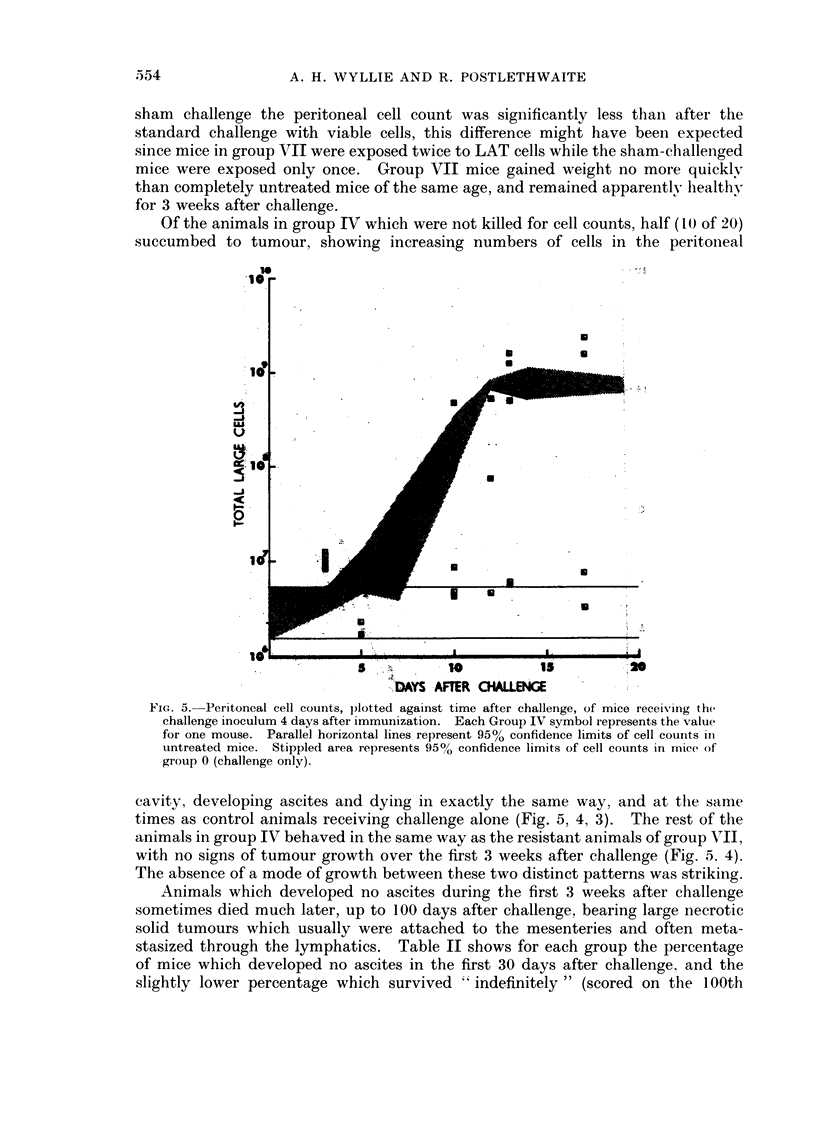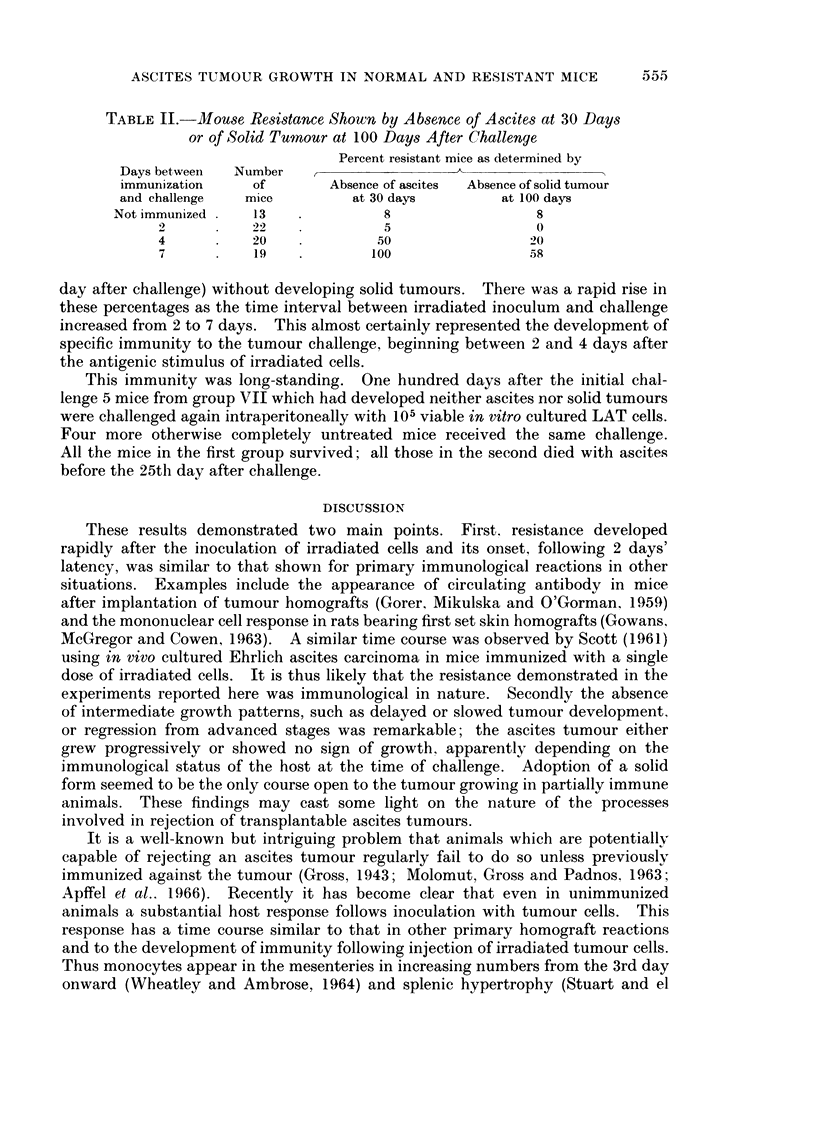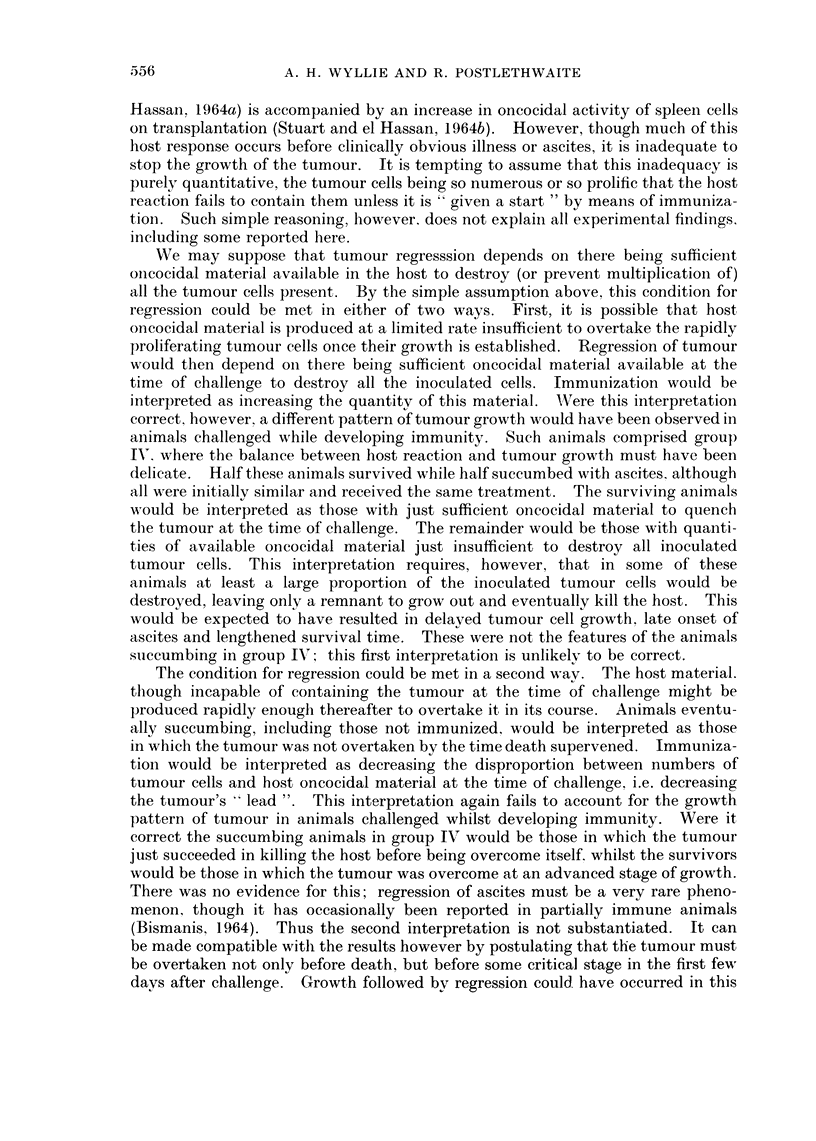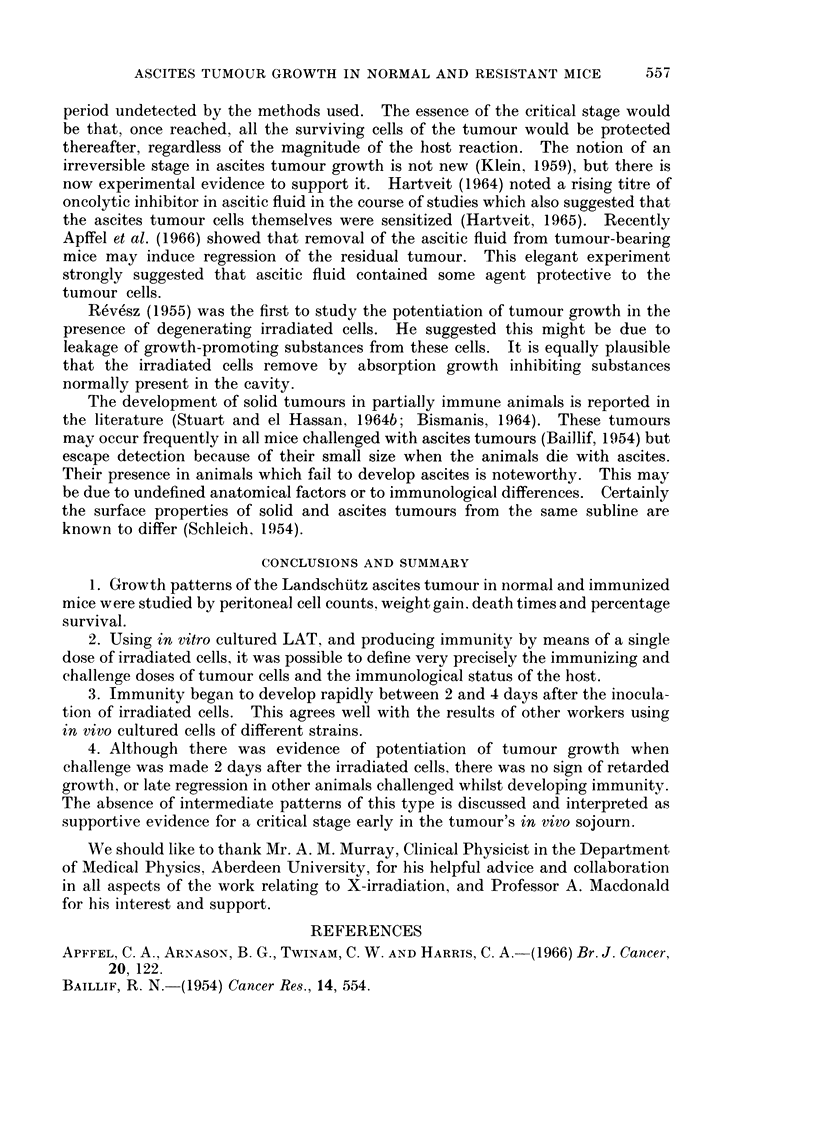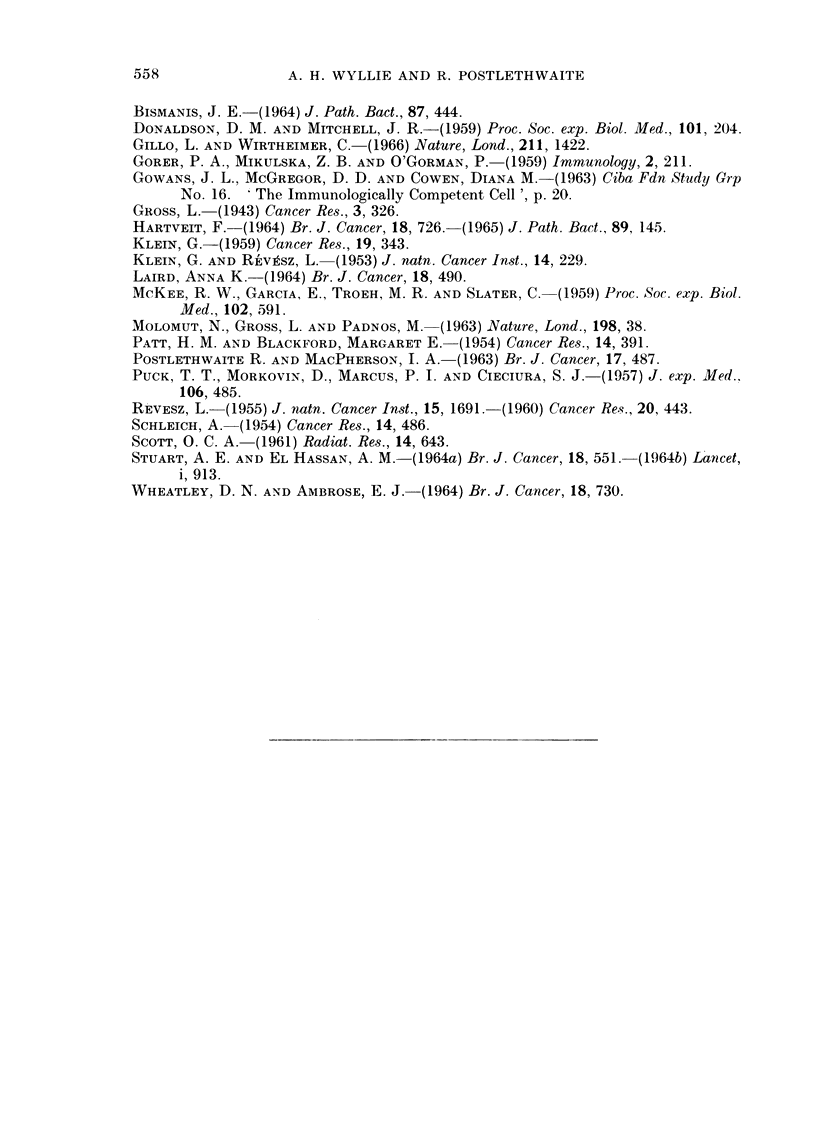# Growth of in vitro cultured Landschütz ascites tumour in normal and resistant mice.

**DOI:** 10.1038/bjc.1967.64

**Published:** 1967-09

**Authors:** A. H. Wyllie, R. Postlethwaite


### GROWTH OF *IN VITRO* CULTURED LANDSCHÜTZ ASCITES TUMOUR IN NORMAL AND RESISTANT MICE

A. H. WYLLIE AND R. POSTLETHWAITE

*From the Department of Bacteriology, Foresterhill, University of Aberdeen*

Received for publication February 3, 1967

THE mode of growth of transplantable ascites tumours can be determined with some accuracy. Tumour cells at any point in time can be easily counted since most of them are free in the peritoneal cavity and so may be withdrawn for study. It has been possible to define clearly the growth pattern of tumours growing from large inocula, in situations where the hosts rapidly succumb (Klein and Révész, 1953; Patt and Blackford, 1954; Laird, 1964). However when the inoculum is small or when the inoculated host is in process of developing immunity the pattern of growth is less certain. When tumour cells are few host cells may outnumber them by some hundreds of times. Moreover slight variations in the size of a small inoculum may mean great differences in the ultimate fate of host and tumour. Using tumour cells obtained directly from animals one can never be certain about the effective size of an inoculum, for not all inoculated cells are viable, particularly if they have been washed carefully beforehand in saline solutions to remove unwanted materials from the previous host (Gillo and Wirtheimer, 1966). Nevertheless a study of tumour behaviour might be instructive in situations where the host need not necessarily succumb, for it is in these situations that host reactions against the tumour are most nearly successful, and perhaps most obvious and easily investigated.

This paper describes growth patterns of the Landschütz ascites tumour (LAT) in normal and resistant mice, using relatively small inocula. It was possible to be fairly certain about the viability of the cells in these inocula since the LAT was maintained *in vitro* before inoculation. Maintained in this way, the cells retained high mouse virulence (Postlethwaite and MacPherson, 1963) while growing nearly exponentially and at a very constant rate. Since they did not adhere to the culture vessel, but floated in free suspension, the tumour cells could be quickly prepared for animal inoculation, without recourse to washing, yet free from contamination from previous hosts. LAT grown *in vitro* proved a plentiful, predictable, easily handled commodity.

The resistant mice received a single large dose of heavily irradiated Landschütz cells. The resistance which develops after inoculation of irradiated cells is generally accepted as being immunological in nature (Donaldson and Mitchell, 1959; McKee *et al.*, 1959; Révész, 1960; Scott, 1961) and in these experiments, involving a long established tumour line of wide host range, the immunity was almost certainly evoked by transplantation antigens (Klein, 1959). The use of a single dose of irradiated cells made it possible to study with some precision the time course of the development of this immunity.

### MATERIALS AND METHODS

*Animals.*—Adult male random-bred TO mice of mean weight 25 g. were fed pellet Diet FFG (E. Dixon & Sons, (Ware) Ltd., Ware, Herts.) and water. They were divided into 4 groups of about 50 mice each, called 0, II, IV and VII. Those in groups II, IV and VII all received $1 \cdot 2 \times 10^6$ heavily irradiated LAT cells by intraperitoneal injection and this was followed by intraperitoneal challenge with $3 \times 10^4$ viable LAT cells. The interval between irradiated inoculum and challenge was 2 days in group II, 4 days in group IV and 7 days in group VII. The control group, group 0, received the challenge alone. $3 \times 10^4$ cells was a suitable size of challenge because it killed about 95% of such control animals. Thus even slight degrees of protection in the other groups were likely to be detected.

The tumour's course was measured (i) by counting the number of cells in the peritoneal cavity; (ii) by weighing the mice daily, for a rough index of the onset and progress of ascites; (iii) by noting the time between challenge and death; and (iv) by noting the percentage of mice surviving in each group.

*Peritoneal cavity cell counts.*—On the 3rd, 5th, 7th, 10th and some subsequent days after challenge, 3 to 5 mice from each group were killed by cervical dislocation. The number of cells in each peritoneal cavity was obtained by multiplying together its cell concentration and its volume. Peritoneal fluid volume was deduced from its mass, found by weighing the carcase before and after carefully swabbing the cavity with cotton wool. It is legitimate to assume a specific gravity of 1·00 for peritoneal fluid for the first 12 days after challenge, the value for a pool of samples from 5 mice being 1·003 on the 12th day. The cell concentration of each peritoneal cavity was obtained by withdrawing 0·1 ml. of fluid into a tuberculin syringe, diluting with 0·1% Trypan Blue in isotonic saline and counting immediately on a Fuchs-Rosenthal chamber. When the volume of peritoneal fluid was very small, 0·1 ml. could not be withdrawn until 1·0 ml. isotonic saline had been injected into the cavity. All punctures of the peritoneum were made through the abdominal wall after reflection of the skin. In every case, before withdrawing the sample for counting the abdomen was massaged between finger and thumb four times per second for 30 seconds.

No attempt was made to differentiate between tumour and host cells save that only the large cells in the counting chamber were considered. Comparison with Giemsa-stained smears showed that the " large cell " count included macrophages and some large lymphocytes as well as tumour cells, but small lymphocytes and polymorphs were excluded. The difference between large and smaller cells was quite distinct. Counts were made of large cells from the peritoneal cavities of 6 entirely untreated mice. The mean value, $3 \cdot 44 \times 10^6$ (SD $= 1 \cdot 88 \times 10^6$), defined the baseline above which tumour growth or host cell reaction could be assessed.

*Cell line.*—LAT cells were grown in 60 mm. glass Petri dishes as described by Postlethwaite and MacPherson (1963). The medium (ETC/80.10.10) consisted of modified Eagle's medium (80%) supplemented with heat-inactivated calf serum (10%), tryptose phosphate broth (10%) and antibiotics (penicillin 100 units per ml.; streptomycin 100 $\mu$g. per ml.; mycostatin 50 $\mu$g. per ml.). Cultures were incubated at 37° C. in an atmosphere of 5% $CO_2$ in air.

When the concentration of cells in a dish approached $10^6$ per ml. the cells were brought into homogeneous suspension by gentle refluxing with a Pasteur pipette. Some were then withdrawn, diluted in fresh medium, and transferred to a new dish at a concentration of $3 \times 10^4$ per ml. By regular passage from dish to dish like this, it was possible to maintain, throughout the period of the experiments, a stock of cells for animal inoculation whose concentration never rose above $2 \times 10^6$ per ml. nor fell below $3 \times 10^4$ per ml. This is the range in which growth is most nearly logarithmic, and very constant (Fig. 1). The mean generation time was 18·8 hr (95% confidence limits 16·5–21·1 hr).

*Irradiation.*—3000 r. were delivered to the cells at 103–106 r. per minute by a therapeutic X-ray machine operating at 230 kv and 15 mA. The beam was

FIG. 1.—Growth of LAT *in vitro* over a period of 55 days, during which cells from this line were used for animal inoculation as described in the text. Horizontal axis: days of *in vitro* growth. Vertical axis: calculated cumulative yield of cells ($\log_{10}$) from " day 0 ", when the cell line was already in its 18th passage *in vitro*. The position of each point was calculated from the cell concentration at the time of passage and the cumulative dilution factor. M.G.T. = Mean generation time.

filtered with 1 mm. Cu and 1 mm. Al. During irradiation the cells were suspended in 20 ml. ETC/80.10.10 in a 10 cm. Petri dish with the lid on, at room temperature and in equilibrium with atmospheric air. For maximum backscatter the dish was placed on a 10 cm. high stack of " Mix-D " blocks. X-ray dose was checked with a Baldwin Farmer Secondary Standard X-ray Dosemeter. 3000 r. did not destroy LAT cells immediately, as checked by exclusion of Trypan Blue, but prevented them from undergoing more than 1 or 2 subsequent divisions. The X-ray sensitivity of LAT growing in glass was shown to be similar to that of other mammalian cells (Puck *et al.*, 1957) with a D37 value of 135 r. This value implies that less than 1 in $10^9$ cells irradiated with 3000 r. would be expected to survive.

*Preparation of inocula.*—All inocula were in 0·2 ml. ETC/80.10.10 and were delivered by a tuberculin syringe. Cell concentration was checked immediately before inoculation and again, in the residue, immediately afterwards. These counts never differed by more than 5%. Challenge inocula could be prepared rapidly, adjustment to the required concentration and inoculation of 50 mice requiring no more than 45 minutes. This was the maximum period during which challenge cells were exposed to room temperature and low $pCO_2$. Irradiated inocula took longer to prepare, but the cells were always inoculated within an hour of the end of irradiation. Irradiation itself never produced a significant immediate change in cell concentration.

### RESULTS

The results showed that, depending on the state of the host at the time of challenge, the tumour may grow progressively or show little evidence of growth at all. There was no evidence for regression of advanced tumour, nor for retarded

FIG. 2.—Peritoneal cell counts, plotted against time after challenge, of mice receiving the challenge inoculum only (Group 0), and of those challenged 2 days (Group II) and 7 days (Group VII) after immunization. Each symbol is the mean count of large cells for 3 to 5 mice of the group indicated. The vertical lines indicate standard deviation and the parallel horizontal lines represent mean cells per untreated mouse with 95% confidence limits. To avoid superimposition some symbols have been shifted slightly laterally on days 3, 5, 7 and 12.

growth of tumour in its ascitic form, but solid tumours appeared late in some animals that failed to develop ascites. The results of peritoneal cavity cell counts for groups 0, II and VII are shown in Fig. 2 and the order of spontaneous deaths for all groups in Fig. 3. Weight gains of mice in all groups are plotted in Fig. 4.

Because of the high background of host cells the peritoneal cell counts were not meaningful up to the 5th day after challenge. Beyond the 12th day after challenge they again ceased to define tumour size since many cells were inaccessible to the method, being bound in loose fibrinous clots within the cavity or infiltrating extensively beyond it. In the meaningful range from the 5th to 12th day there was a clear difference between the cell counts from group 0 animals (challenge only) and those from group II animals (challenge 2 days after irradiated cells). On the 10th day after challenge this difference amounted to 300 million more cells per cavity in group II mice, more than a two-fold excess. It was

FIG. 3.—Percentage survival of mice plotted against time after challenge. Only those mice are considered which were not killed deliberately for peritoneal cell counts, and no such executions were carried out in any group after the onset of spontaneous deaths.

○ = Group 0: challenge only (13 mice)
▲ = Group II: challenge 2 days after immunization (22 mice)
◪ = Group IV: challenge 4 days after immunization (20 mice)
● = Group VII: challenge 7 days after immunization (19 mice)

probably a genuine difference in tumour cell growth, and contributed to the shorter survival time of the animals in group II. The onset of ascites, as indicated by rapid weight gain, occurred in both groups at the same time: the 11th day after challenge.

Group VII animals never showed convincing evidence of tumour cell growth. The small rise in number of peritoneal cavity cells seen in Fig. 2 probably represented an increase in host macrophages and large lymphocytes, since it occurred to a similar extent in animals given a sham challenge of medium ETC/80.10.10 only, 7 days after the irradiated inoculum (Table I). Although on the 5th day after

TABLE I.—*Peritoneal Cell Counts in Mice of Group VII and in Similarly Immunised Mice Challenged After 7 Days With Medium Only*

| Days after challenge | Mean total large cells × $10^6$ Group VII | Sham | P (t test) |
| --- | --- | --- | --- |
| 3 | 9·46 (4*) | 9·56 (4) | 0·9–0·95 |
| 5 | 16·00 (5) | 9·06 (4) | <0·001 |
| 7 | 14·75 (5) | 11·50 (3) | 0·4–0·5 |

* Figures in brackets indicate numbers of mice.

FIG. 4.—Mean increase in mouse weight plotted against time after challenge

■ completely untreated mice
○ Group 0: challenge only
▲ Group II: challenge 2 days after immunization
● Group VII: challenge 7 days after immunization
◩ Group IV: challenge 4 days after immunization. Those mice in this group which eventually developed ascites are plotted separately (. . . . . . . . . . . .) from those which did not (– – – – – – – – – –)

To simplify the diagram the results between days 3 and 10 are plotted for Group IV only. The other groups gave closely similar results during this period. Standard deviations were proportionately similar for all points and have been shown (vertical bars) for Group IV mice only on the 18th day after challenge. The baseline (0 weight increase) is the mean weight for the mice in each group measured over a period of 5 days immediately following challenge.

sham challenge the peritoneal cell count was significantly less than after the standard challenge with viable cells, this difference might have been expected since mice in group VII were exposed twice to LAT cells while the sham-challenged mice were exposed only once. Group VII mice gained weight no more quickly than completely untreated mice of the same age, and remained apparently healthy for 3 weeks after challenge.

Of the animals in group IV which were not killed for cell counts, half (10 of 20) succumbed to tumour, showing increasing numbers of cells in the peritoneal

Animals which developed no ascites during the first 3 weeks after challenge sometimes died much later, up to 100 days after challenge, bearing large necrotic solid tumours which usually were attached to the mesenteries and often metastasized through the lymphatics. Table II shows for each group the percentage of mice which developed no ascites in the first 30 days after challenge, and the slightly lower percentage which survived "indefinitely" (scored on the 100th

TABLE II.—*Mouse Resistance Shown by Absence of Ascites at* 30 *Days or of Solid Tumour at* 100 *Days After Challenge*

| Days between immunization and challenge | Number of mice | Percent resistant mice as determined by: Absence of ascites at 30 days | Absence of solid tumour at 100 days |
|---|---|---|---|
| Not immunized | 13 | 8 | 8 |
| 2 | 22 | 5 | 0 |
| 4 | 20 | 50 | 20 |
| 7 | 19 | 100 | 58 |

day after challenge) without developing solid tumours. There was a rapid rise in these percentages as the time interval between irradiated inoculum and challenge increased from 2 to 7 days. This almost certainly represented the development of specific immunity to the tumour challenge, beginning between 2 and 4 days after the antigenic stimulus of irradiated cells.

This immunity was long-standing. One hundred days after the initial challenge 5 mice from group VII which had developed neither ascites nor solid tumours were challenged again intraperitoneally with $10^5$ viable *in vitro* cultured LAT cells. Four more otherwise completely untreated mice received the same challenge. All the mice in the first group survived; all those in the second died with ascites before the 25th day after challenge.

### DISCUSSION

These results demonstrated two main points. First, resistance developed rapidly after the inoculation of irradiated cells and its onset, following 2 days' latency, was similar to that shown for primary immunological reactions in other situations. Examples include the appearance of circulating antibody in mice after implantation of tumour homografts (Gorer, Mikulska and O'Gorman, 1959) and the mononuclear cell response in rats bearing first set skin homografts (Gowans, McGregor and Cowen, 1963). A similar time course was observed by Scott (1961) using *in vivo* cultured Ehrlich ascites carcinoma in mice immunized with a single dose of irradiated cells. It is thus likely that the resistance demonstrated in the experiments reported here was immunological in nature. Secondly the absence of intermediate growth patterns, such as delayed or slowed tumour development, or regression from advanced stages was remarkable; the ascites tumour either grew progressively or showed no sign of growth, apparently depending on the immunological status of the host at the time of challenge. Adoption of a solid form seemed to be the only course open to the tumour growing in partially immune animals. These findings may cast some light on the nature of the processes involved in rejection of transplantable ascites tumours.

It is a well-known but intriguing problem that animals which are potentially capable of rejecting an ascites tumour regularly fail to do so unless previously immunized against the tumour (Gross, 1943; Molomut, Gross and Padnos, 1963; Apffel *et al.*, 1966). Recently it has become clear that even in unimmunized animals a substantial host response follows inoculation with tumour cells. This response has a time course similar to that in other primary homograft reactions and to the development of immunity following injection of irradiated tumour cells. Thus monocytes appear in the mesenteries in increasing numbers from the 3rd day onward (Wheatley and Ambrose, 1964) and splenic hypertrophy (Stuart and el

Hassan, 1964*a*) is accompanied by an increase in oncocidal activity of spleen cells on transplantation (Stuart and el Hassan, 1964*b*). However, though much of this host response occurs before clinically obvious illness or ascites, it is inadequate to stop the growth of the tumour. It is tempting to assume that this inadequacy is purely quantitative, the tumour cells being so numerous or so prolific that the host reaction fails to contain them unless it is " given a start " by means of immunization. Such simple reasoning, however, does not explain all experimental findings, including some reported here.

We may suppose that tumour regresssion depends on there being sufficient oncocidal material available in the host to destroy (or prevent multiplication of) all the tumour cells present. By the simple assumption above, this condition for regression could be met in either of two ways. First, it is possible that host oncocidal material is produced at a limited rate insufficient to overtake the rapidly proliferating tumour cells once their growth is established. Regression of tumour would then depend on there being sufficient oncocidal material available at the time of challenge to destroy all the inoculated cells. Immunization would be interpreted as increasing the quantity of this material. Were this interpretation correct, however, a different pattern of tumour growth would have been observed in animals challenged while developing immunity. Such animals comprised group IV, where the balance between host reaction and tumour growth must have been delicate. Half these animals survived while half succumbed with ascites, although all were initially similar and received the same treatment. The surviving animals would be interpreted as those with just sufficient oncocidal material to quench the tumour at the time of challenge. The remainder would be those with quantities of available oncocidal material just insufficient to destroy all inoculated tumour cells. This interpretation requires, however, that in some of these animals at least a large proportion of the inoculated tumour cells would be destroyed, leaving only a remnant to grow out and eventually kill the host. This would be expected to have resulted in delayed tumour cell growth, late onset of ascites and lengthened survival time. These were not the features of the animals succumbing in group IV; this first interpretation is unlikely to be correct.

The condition for regression could be met in a second way. The host material, though incapable of containing the tumour at the time of challenge might be produced rapidly enough thereafter to overtake it in its course. Animals eventually succumbing, including those not immunized, would be interpreted as those in which the tumour was not overtaken by the time death supervened. Immunization would be interpreted as decreasing the disproportion between numbers of tumour cells and host oncocidal material at the time of challenge, i.e. decreasing the tumour's " lead ". This interpretation again fails to account for the growth pattern of tumour in animals challenged whilst developing immunity. Were it correct the succumbing animals in group IV would be those in which the tumour just succeeded in killing the host before being overcome itself, whilst the survivors would be those in which the tumour was overcome at an advanced stage of growth. There was no evidence for this; regression of ascites must be a very rare phenomenon, though it has occasionally been reported in partially immune animals (Bismanis, 1964). Thus the second interpretation is not substantiated. It can be made compatible with the results however by postulating that the tumour must be overtaken not only before death, but before some critical stage in the first few days after challenge. Growth followed by regression could have occurred in this period undetected by the methods used. The essence of the critical stage would be that, once reached, all the surviving cells of the tumour would be protected thereafter, regardless of the magnitude of the host reaction. The notion of an irreversible stage in ascites tumour growth is not new (Klein, 1959), but there is now experimental evidence to support it. Hartveit (1964) noted a rising titre of oncolytic inhibitor in ascitic fluid in the course of studies which also suggested that the ascites tumour cells themselves were sensitized (Hartveit, 1965). Recently Apffel *et al.* (1966) showed that removal of the ascitic fluid from tumour-bearing mice may induce regression of the residual tumour. This elegant experiment strongly suggested that ascitic fluid contained some agent protective to the tumour cells.

Révész (1955) was the first to study the potentiation of tumour growth in the presence of degenerating irradiated cells. He suggested this might be due to leakage of growth-promoting substances from these cells. It is equally plausible that the irradiated cells remove by absorption growth inhibiting substances normally present in the cavity.

The development of solid tumours in partially immune animals is reported in the literature (Stuart and el Hassan, 1964*b*; Bismanis, 1964). These tumours may occur frequently in all mice challenged with ascites tumours (Baillif, 1954) but escape detection because of their small size when the animals die with ascites. Their presence in animals which fail to develop ascites is noteworthy. This may be due to undefined anatomical factors or to immunological differences. Certainly the surface properties of solid and ascites tumours from the same subline are known to differ (Schleich, 1954).

### CONCLUSIONS AND SUMMARY

1. Growth patterns of the Landschütz ascites tumour in normal and immunized mice were studied by peritoneal cell counts, weight gain, death times and percentage survival.

2. Using *in vitro* cultured LAT, and producing immunity by means of a single dose of irradiated cells, it was possible to define very precisely the immunizing and challenge doses of tumour cells and the immunological status of the host.

3. Immunity began to develop rapidly between 2 and 4 days after the inoculation of irradiated cells. This agrees well with the results of other workers using *in vivo* cultured cells of different strains.

4. Although there was evidence of potentiation of tumour growth when challenge was made 2 days after the irradiated cells, there was no sign of retarded growth, or late regression in other animals challenged whilst developing immunity. The absence of intermediate patterns of this type is discussed and interpreted as supportive evidence for a critical stage early in the tumour's *in vivo* sojourn.

We should like to thank Mr. A. M. Murray, Clinical Physicist in the Department of Medical Physics, Aberdeen University, for his helpful advice and collaboration in all aspects of the work relating to X-irradiation, and Professor A. Macdonald for his interest and support.

### REFERENCES

APFFEL, C. A., ARNASON, B. G., TWINAM, C. W. AND HARRIS, C. A.—(1966) *Br. J. Cancer*, **20**, 122.
BAILLIF, R. N.—(1954) *Cancer Res.*, **14**, 554.

BISMANIS, J. E.—(1964) *J. Path. Bact.*, **87**, 444.
DONALDSON, D. M. AND MITCHELL, J. R.—(1959) *Proc. Soc. exp. Biol. Med.*, **101**, 204.
GILLO, L. AND WIRTHEIMER, C.—(1966) *Nature, Lond.*, **211**, 1422.
GORER, P. A., MIKULSKA, Z. B. AND O'GORMAN, P.—(1959) *Immunology*, **2**, 211.
GOWANS, J. L., MCGREGOR, D. D. AND COWEN, DIANA M.—(1963) *Ciba Fdn Study Grp* No. 16. 'The Immunologically Competent Cell', p. 20.
GROSS, L.—(1943) *Cancer Res.*, **3**, 326.
HARTVEIT, F.—(1964) *Br. J. Cancer*, **18**, 726.—(1965) *J. Path. Bact.*, **89**, 145.
KLEIN, G.—(1959) *Cancer Res.*, **19**, 343.
KLEIN, G. AND RÉVÉSZ, L.—(1953) *J. natn. Cancer Inst.*, **14**, 229.
LAIRD, ANNA K.—(1964) *Br. J. Cancer*, **18**, 490.
MCKEE, R. W., GARCIA, E., TROEH, M. R. AND SLATER, C.—(1959) *Proc. Soc. exp. Biol. Med.*, **102**, 591.
MOLOMUT, N., GROSS, L. AND PADNOS, M.—(1963) *Nature, Lond.*, **198**, 38.
PATT, H. M. AND BLACKFORD, MARGARET E.—(1954) *Cancer Res.*, **14**, 391.
POSTLETHWAITE R. AND MACPHERSON, I. A.—(1963) *Br. J. Cancer*, **17**, 487.
PUCK, T. T., MORKOVIN, D., MARCUS, P. I. AND CIECIURA, S. J.—(1957) *J. exp. Med.*, **106**, 485.
REVESZ, L.—(1955) *J. natn. Cancer Inst.*, **15**, 1691.—(1960) *Cancer Res.*, **20**, 443.
SCHLEICH, A.—(1954) *Cancer Res.*, **14**, 486.
SCOTT, O. C. A.—(1961) *Radiat. Res.*, **14**, 643.
STUART, A. E. AND EL HASSAN, A. M.—(1964*a*) *Br. J. Cancer*, **18**, 551.—(1964*b*) *Lancet*, i, 913.
WHEATLEY, D. N. AND AMBROSE, E. J.—(1964) *Br. J. Cancer*, **18**, 730.